# National Trends in Gender Representation, Segregation, and Disparities in the Saudi Dental Workforce, 2016–2025

**DOI:** 10.1016/j.identj.2026.111091

**Published:** 2026-08-17

**Authors:** Fahad BaHammam, Hussam M. Alqahtani, Fathima Farook

**Affiliations:** a Department of Restorative and Prosthetic Dental Sciences, College of Dentistry, King Saud bin Abdulaziz University for Health Sciences, Riyadh, Saudi Arabia; b Department of Preventive Dental Sciences, College of Dentistry, King Saud bin Abdulaziz University for Health Sciences, Riyadh, Saudi Arabia; c King Abdullah International Medical Research Centre, Riyadh, Saudi Arabia; d Dental Services, King Abdulaziz Medical City, Ministry of National Guard - Health Affairs, Riyadh, Saudi Arabia

**Keywords:** Gender equity, Saudi Arabia, Workforce diversity, Workforce, Dentists, Demographics

## Abstract

**Introduction and aims:**

Despite the importance of gender equity for promoting fairness and informing workforce planning, key indicators of gender equity in the Saudi dental workforce remain underexplored. This study assessed gender representation in the Saudi dental workforce from 2016 to 2025, including gender segregation and disparities across specialties and professional ranks.

**Methods:**

This study analysed Saudi Commission for Health Specialties registry data from 2016 to 2025. Vertical and horizontal gender segregation were assessed using the Local Disproportionality Index (LDI), based on the female representation within each professional rank and specialty relative to the overall female proportion in the workforce for each year. Gender disparities were examined for each year using proportional-odds ordinal logistic regression to assess the association between gender and professional rank, and weighted multinomial logistic regression to assess the association between gender and specialty registration. Models were adjusted for age group, nationality, and country of qualification.

**Results:**

Based on LDI values, female dentists were relatively overrepresented in lower professional ranks and underrepresented in higher ranks. Compared with male dentists, female dentists had lower odds of being represented in a higher professional rank across all years, although the adjusted odds ratio increased from 0.48 (95% CI, 0.45-0.51) in 2016 to 0.67 (95% CI, 0.64-0.70) in 2025. Across specialties, female dentists were underrepresented in most specialties, particularly surgical specialties, but were overrepresented in a few nonclinical specialties and paediatric dentistry. Female representation increased over time across most specialties. These patterns were broadly consistent with adjusted weighted multinomial logistic regression.

**Conclusion:**

The Saudi dental workforce exhibited persistent gender disparities over the past decade, including both horizontal and vertical gender segregation. Although these patterns appeared to become less pronounced over time, they still have important implications for workforce planning, service organisation, and access to dental care in Saudi Arabia.

## Introduction

Gender equity refers to fairness in access to professional opportunities, resources, and career advancement, such that individuals are not disadvantaged by gender-related barriers, bias, or discrimination.^1^ It is widely recognised as an important concept in dental workforce planning and policymaking because it can enhance innovation and creativity within the profession by enabling a broader range of perspectives and problem-solving approaches, particularly at the leadership level.^2^ Gender equity may also foster patient trust and improve clinical communication by supporting a workforce that better reflects the diversity of the patient population.^3^ In addition, it may reduce occupational dissatisfaction and attrition, thereby helping to retain qualified dentists.^4^ Despite its importance, gender equity and its related concepts have rarely been investigated in relation to the dental workforce in Saudi Arabia.^5^^,^^6^

Although gender equity extends beyond gender representation in the dental workforce, gender representation may serve as a useful starting point for assessing gender equity within the workforce.^7^ Globally, there has been an increase in female representation in the dental workforce over recent decades.^7^ In fact, in many countries, women now constitute approximately half of the dental profession.^7^ However, this has not always translated into equal representation in senior positions or across various dental specialties.^2^^,^^4^^,^^8^ This may indicate that, despite achieving numerical parity at the aggregate level, the dental workforce in many countries has yet to achieve gender equity due to the persistence of both vertical and horizontal gender segregation. Alqahtani et al. (2022) analysed trends in the composition of the dental workforce in Saudi Arabia and reported that women constituted approximately 45% of the workforce.^9^ Their findings also suggested the possible presence of both horizontal and vertical gender segregation.^9^ These findings are consistent with more recent studies examining workforce composition across specific dental specialties in Saudi Arabia.^10^, ^11^, ^12^, ^13^

The patterns of vertical and horizontal gender segregation may be influenced by factors other than those directly related to gender. For instance, in many workforces, female dentists tend to be younger with fewer years of experience and consequently be less likely to occupy senior positions.^14^ Another potential confounding factor is workload intensity, as female dentists may work fewer hours on average over the course of their careers.^15^ In addition, differences between male and female dentists in practice ownership and employment sector have been suggested as additional potential confounding factors.^16^ Adjusting for these confounding factors is important for identifying true gender-related disparities, which serve as a key indicator of gender equity within the dental workforce.^17^

In light of these considerations, this study aimed to assess gender representation in the dental workforce in Saudi Arabia from 2016 to 2025, including temporal patterns of segregation and disparities across specialties and professional ranks. This study addresses an important gap in the literature on gender equity within the Saudi dental workforce and its findings could inform workforce planning and policymaking aimed at advancing gender equity in Saudi Arabia.

## Methods

### Ethical consideration

The study complied with the Saudi National Committee of Bioethics regulations and received Institutional Review Board (IRB) approval from the King Abdullah International Medical Research Centre (IRB# NRR26/045/2). The analysis used de-identified SCFHS registry data, and the Institutional Review Board (IRB) of the King Abdullah International Medical Research Centre (IRB# NRR26/045/2) granted a waiver of informed consent.

### Study design and data source

This retrospective, registry-based study analysed year-level aggregate data extracted in July 2025 from the Saudi Commission for Health Specialties (SCFHS) registry for the period 2016–2025. The registry provided annual counts of all licensed dentists practicing in Saudi Arabia, stratified by gender, professional rank, specialty, nationality, age group, and country of qualification, providing a complete cross-sectional snapshot of the workforce for each year. No missing observations were present within the available registry variables used in the analysis. However, data for the training-resident category were unavailable before 2017 because SCFHS registration for training residents was not mandatory before that year.

#### Variables and definitions

The operational definitions of the study variables were based primarily on the SCFHS registry definitions, with modifications made where necessary. In the SCFHS registry, gender is self-reported by the licensed dentist and is recorded as a binary variable (male or female). Professional rank is classified into 6 ascending categories, from General Dentist to Consultant (Table 1).^11^^,^^18^ Nationality is recorded as Saudi or non-Saudi. Age is categorised into 5-year groups. In this study, dentists aged younger than 30 years and 60 years or older were each combined into a single category. Country of qualification refers to the country in which the dentist obtained the highest dental qualification recognised by SCFHS for professional rank classification. In this study, countries of qualification were categorised as either Saudi Arabia or international.

**Table 1:** Professional rank classification of dentists according to the SCFHS.

Rank	Description
Consultant	A dentist who holds a certificate from the first or second group, has the required experience for the consultant level, and has completed all other classification requirements.
Senior Registrar	A dentist who holds a certificate from the first or second group and has not completed the required experience for the rank of consultant.
Registrar	A dentist who holds a certificate from one of the third group certificates and has completed all other classification requirements.
Resident	A dentist who holds a certificate from one of the third group certificates and has not completed the required experience for the rank of registrar.
Training Resident	A dental student enrolled in a postgraduate training program in Saudi Arabia*
General Dentist	A dentist who holds a bachelor's degree in dentistry from a recognised dental college in the country of origin and has completed all other classification requirements.

⁎ In the context of the SCFHS registry, this category refers to dentists enrolled in postgraduate dental training programs and does not refer to undergraduate dental students. Reproduced from BaHammam and Alqahtani (2025), published in BMC Oral Health (ISSN 1472-6831). Licensed under CC BY 4.0. DOI: 10.1186/s12903-025-07354-8.

Because the SCFHS does not publish an official list of recognised dental specialties, the specialty labels recorded in the SCFHS registry were not fully consistent. Therefore, these labels were thematically harmonised by 2 independent researchers (FB and HA) using a hierarchical mapping approach. First, labels were matched to the names of Saudi Board dental training programs. If no match was identified, they were mapped to dental specialties recognised by the American Dental Association. Labels that could not be matched were assigned the most commonly used label in the registry. Ambiguous or overlapping labels were reviewed independently and classified according to the primary specialty component represented in the label. Mapping disagreements were resolved through discussion between the 2 researchers, involving a third researcher (FF) when necessary. The mapping of original SCFHS specialty labels to harmonised specialty categories is shown in Supplementary Table S1. Only specialties that were represented in the registry for at least 8 years during the study period and had at least 5 practitioners per year were included. This resulted in 18 eligible dental specialties, in addition to General Dentistry.^11^^,^^18^

### Statistical analysis

The annual percentage of female dentists in the Saudi dental workforce was calculated for each year from 2016 to 2025 as (number of female dentists ÷ total number of dentists) × 100. Linear regression was then employed to quantify the temporal trend in female dentist representation over the study period, with calendar year entered as a continuous predictor and the annual percentage of female dentists as the outcome variable.

The annual representation of female dentists within each professional rank and dental specialty, relative to the overall female proportion in the dental workforce for the same year, was assessed using the Local Disproportionality Index (LDI) to evaluate patterns of vertical and horizontal gender segregation within the Saudi dental workforce. For each rank and specialty, the LDI was calculated annually as the proportion of female dentists in that rank or specialty divided by the proportion of female dentists in the overall dental workforce, as shown in Equations (1) and (2). An LDI greater than 1 indicated relative female overrepresentation within that rank or specialty, whereas an LDI below 1 indicated relative underrepresentation. Temporal patterns were visualised using rank-by-year and specialty-by-year heatmaps, with colour intensity encoding the magnitude of deviation from parity (LDI = 1).

(1) $$\text{LD}I_{r,t}=\frac{\frac{F_{r,t}}{T_{r,t}}}{\frac{F_{t}}{T_{t}}}$$

• $F_{r,t}$: Number of female dentists in rank r in year t
• $T_{r,t}$: Number of dentists in rank r in year t
• $F_{t}$: Number of female dentists in the workforce in year t
• $T_{t}$: Total number of dentists in the workforce in year t

Equation (1). LDI for professional rank.

(2) $$\text{LD}I_{s,t}=\frac{\frac{F_{s,t}}{T_{s,t}}}{\frac{F_{t}}{T_{t}}}$$

• $F_{s,t}:$ Number of female dentists in specialty s in year t
• $T_{s,t}:$ Number of dentists in specialty s in year t
• $F_{t}:$ Number of female dentists in the workforce in year t
• $T_{t}:$ Total number of dentists in the workforce in year t

Equation (2). LDI for dental specialty.

Proportional-odds ordinal logistic regression models were fitted separately for each year (2016–2025) to examine the association between gender and professional rank. Professional rank was modelled as an ordered outcome with 6 ascending categories: General Dentist, Training Resident, Resident, Registrar, Senior Registrar, and Consultant. The primary predictor of interest was gender (male vs. female). Age group, nationality, and country of qualification were included as covariates. Given that the dataset consisted of aggregated counts, the number of dentists represented by each record was used as frequency weights in the model. Adjusted odds ratios (ORs) with 95% confidence intervals (CIs) were estimated using Wald statistics and represent the odds of being in higher professional rank.

The proportional-odds assumption was assessed using a likelihood-ratio test comparing the proportional-odds model with a partial-proportional-odds model. The tests indicated significant violations in all annual models (all p < 0.001). However, because likelihood-ratio tests are highly sensitive in large samples and may detect minor departures of limited practical importance.^19^^,^^20^ we examined the gender coefficients across the separate cumulative binary regressions and found them to be broadly similar across rank thresholds. We therefore retained the proportional-odds model for parsimony and interpret the odds ratios as summary estimates of the association between gender and professional rank across cumulative thresholds, rather than as identical effects at each threshold.

Weighted multinomial logistic regression models were fitted separately for each year (2016–2025) to estimate the association between gender and dental specialty registration among licensed dentists, adjusting for age group, nationality, and country of qualification. General Dentistry was set as the reference category. Adjusted odds ratios (ORs) and 95% confidence intervals (CIs) were calculated. In the SCFHS registry, dentists cannot hold more than one major specialty registration simultaneously. Therefore, each dentist was assigned to a single specialty category within each annual workforce snapshot, and the specialty outcome categories used in the multinomial logistic regression were mutually exclusive.

Regression models were fitted separately for each year because the registry data consisted of aggregated annual counts rather than individual-level longitudinal records. Although each year represented a complete cross-sectional snapshot of licensed dentists, the same dentist could appear in the registry across multiple years, either in the same category or after moving to another professional rank or specialty category. Because individual dentists could not be linked across years, a pooled repeated cross-sectional model would risk treating annual observations as independent person-year records and would require assumptions about within-person correlation and transitions over time that could not be assessed using the available aggregated data. Therefore, separate annual models were considered more appropriate for describing year-specific gender-related patterns within each cross-sectional snapshot.

All regression analyses were weighted by the number of dentists to account for the aggregated nature of the registry data. The regression model results were used to assess gender disparities across specialties and professional ranks. All analyses in this study were performed using R version 4.3.0 with the R packages tidyverse, nnet, and MASS.

## Results

### Overall trends in annual gender composition (2016–2025)

**Fig. 1:**
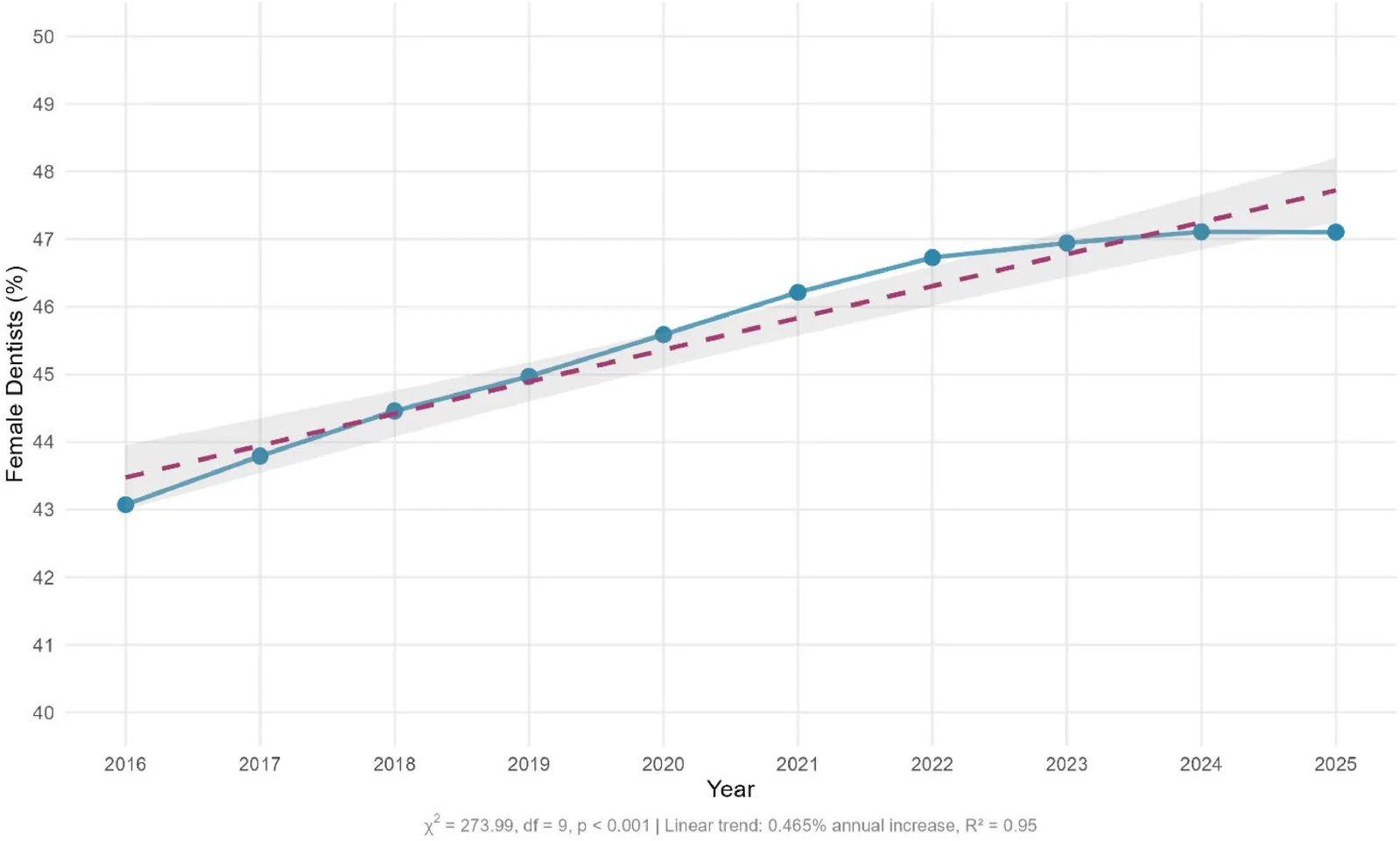
Trends in female representation in the dental workforce in Saudi Arabia, 2016–2025.

While male dentists composed the majority of the Saudi dental workforce throughout the study period (2016–2025), the percentage of female dentists steadily increased from 43.1% (11,536 of 26,783) in 2016 to 47.1% (24,141 of 51,250) in 2025, representing a 4.0 percentage point increase (Table 2). Notably, after 2022, the rate of increase in female representation slowed, with female representation peaking at 47.1% in 2024–2025. Linear regression demonstrated a significant annual increase of 0.465 percentage points in female representation (R² = 0.95) (Figure 1).

**Table 2:** Annual gender composition of the dental workforce in Saudi Arabia, 2016–2025.

Year	Total n	Female n (%)	Male n (%)
2016	26,783	11,536 (43.1%)	15,247 (56.9%)
2017	29,636	12,978 (43.8%)	16,658 (56.2%)
2018	32,189	14,311 (44.5%)	17,878 (55.5%)
2019	35,044	15,759 (45.0%)	19,285 (55.0%)
2020	37,951	17,301 (45.6%)	20,650 (54.4%)
2021	41,377	19,122 (46.2%)	22,255 (53.8%)
2022	44,319	20,709 (46.7%)	23,610 (53.3%)
2023	47,212	22,163 (46.9%)	25,049 (53.1%)
2024	50,305	23,698 (47.1%)	26,607 (52.9%)
2025	51,250	24,141 (47.1%)	27,109 (52.9%)

### Gender segregation and disparities by professional rank

**Fig. 2:**
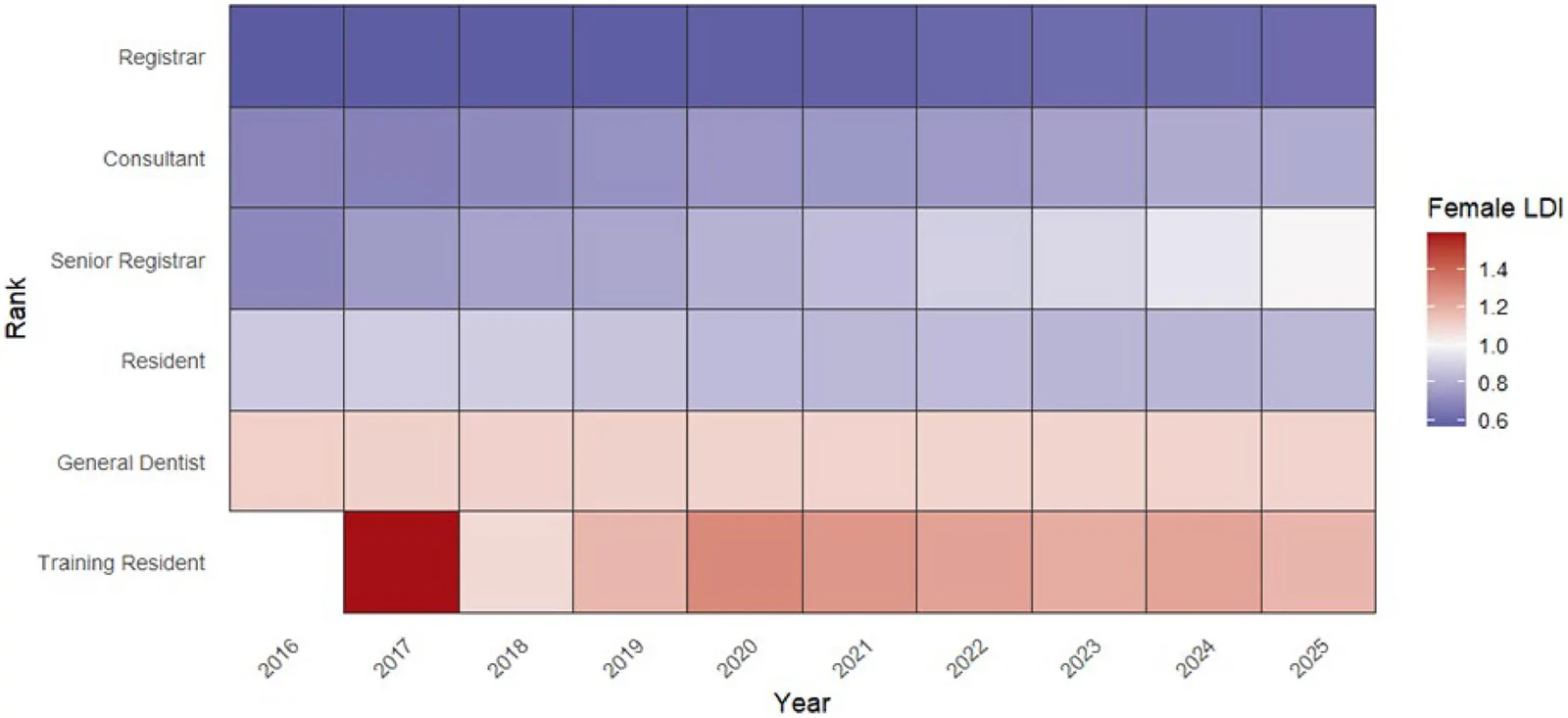
Heatmap of female relative representation, based on LDI values, across professional ranks in Saudi Arabia, 2016–2025; LDI = 1 indicates proportional representation, LDI > 1 female overrepresentation, and LDI < 1 underrepresentation.

Based on the LDI values, female dentists were relatively overrepresented in lower professional ranks (General Dentist and Training Resident) and relatively underrepresented in higher ranks (Figure 2 and Supplementary Table S2). Overall, the relative representation of female dentists within the General Dentist rank did not change substantially over the 10-year period. However, the 3 highest professional ranks (Consultant, Senior Registrar, and Registrar) showed some improvement in female relative representation, most notably among Senior Registrars, who reached approximately proportional representation in 2025. In contrast, female relative representation in the Resident rank decreased slightly over the 10-year period. Finally, while female dentists were relatively overrepresented in the Training Resident rank, their relative representation fluctuated markedly across years.

The proportional-odds ordinal logistic regression models, adjusted for age group, nationality, and country of qualification, showed that female dentists had consistently lower odds of being represented in higher professional ranks than male dentists across all years (Table 3). The odds ratio for female dentists increased modestly from 0.48 (95% CI: 0.45–0.51) in 2016 to 0.67 (95% CI: 0.64–0.70) in 2025, remaining unchanged from 2023 onward.

**Table 3:** Adjusted odds ratios for female dentists being represented in higher professional ranks compared with male dentists, by year, 2016–2025.

Year	OR (95% CI)
2016	0.48 (0.45-0.51)
2017	0.50 (0.47-0.53)
2018	0.52 (0.49-0.55)
2019	0.55 (0.52-0.58)
2020	0.60 (0.57-0.63)
2021	0.62 (0.59-0.65)
2022	0.65 (0.62-0.67)
2023	0.67 (0.64-0.70)
2024	0.67 (0.64-0.69)
2025	0.67 (0.64-0.70)

### Gender segregation and disparities by dental specialty

**Fig. 3:**
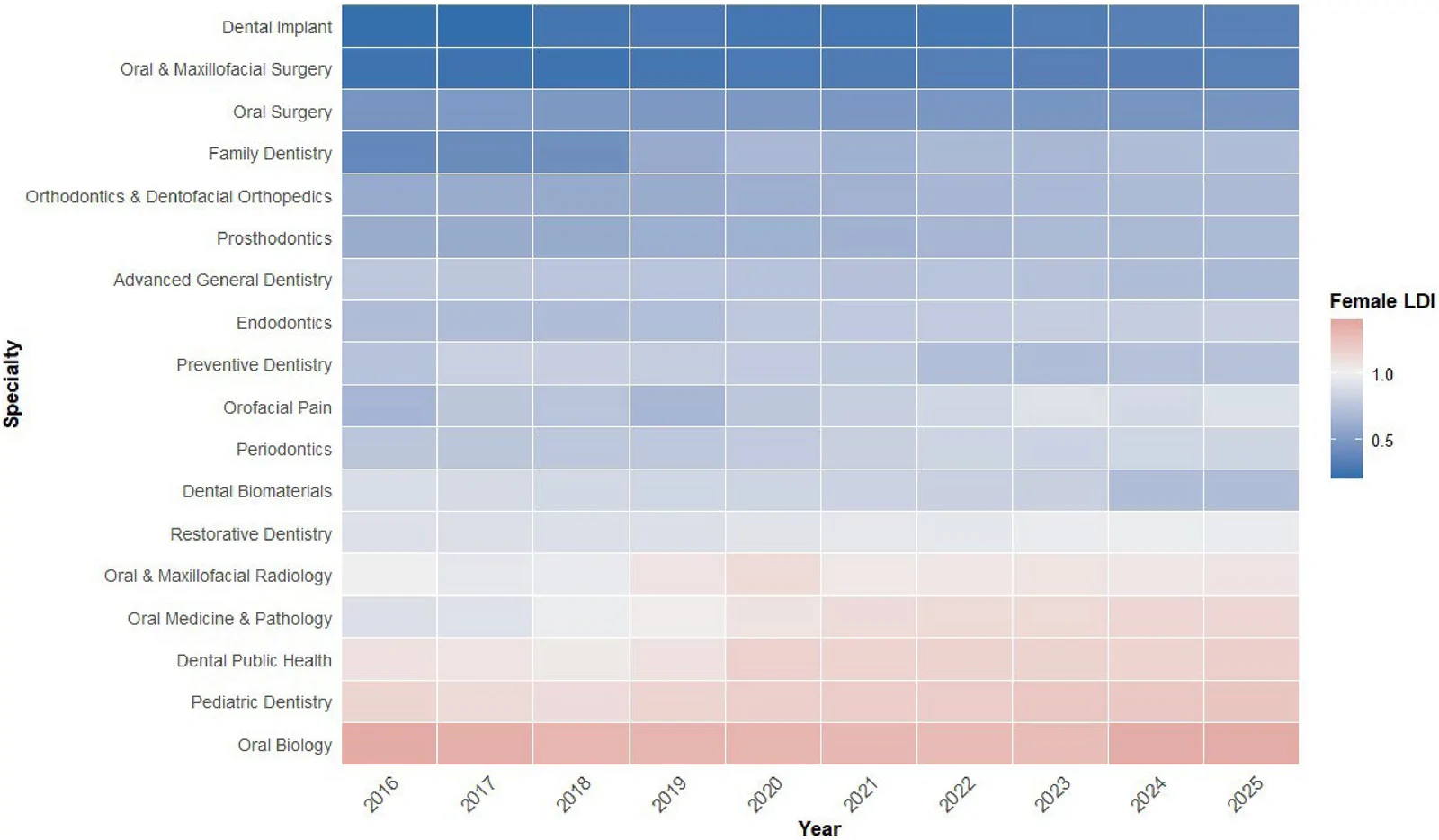
Heatmap of female relative representation, based on LDI values, across dental specialties in Saudi Arabia, 2016–2025; LDI = 1 indicates proportional representation, LDI > 1 female overrepresentation, and LDI < 1 underrepresentation.

By 2025, among the 18 dental specialties, female dentists were relatively overrepresented in only 5 specialties: Oral and Maxillofacial Radiology, Oral Medicine and Pathology, Dental Public Health, Paediatric Dentistry, and Oral Biology (Figure 3 and Supplementary Table S3). These specialties were also the only ones that showed higher adjusted odds of female (vs. male) registration after adjustment for age group, nationality, and country of qualification, based on the weighted multinomial logistic regression results (with General Dentistry as the reference category) (Table 4). Oral Biology showed the greatest relative overrepresentation and the highest adjusted odds ratio in 2025 (OR = 2.52, 95% CI: 1.16-5.49). In contrast, the adjusted odds ratio for Oral and Maxillofacial Radiology was the lowest at 1.26 (95% CI: 0.78-2.06) and was the only estimate whose 95% CI included 1. Most of these 5 specialties showed overall increases in female relative representation over the 10-year period, with Oral Medicine and Pathology demonstrating the most pronounced increase. In contrast, the relative representation of female dentists in Oral Biology remained largely stable. The adjusted odds ratio point estimates for the 5 specialties were also generally higher in later years, although the 95% CIs overlapped across years.

**Table 4:** Adjusted odds ratios with 95% CIs for female versus male dentists being registered in dental specialties, by year, 2016–2025.

Specialty	2016	2017	2018	2019	2020	2021	2022	2023	2024	2025
Oral & Maxillofacial Surgery	0.15 (0.12-0.19)	0.15 (0.12-0.19)	0.15 (0.12-0.19)	0.17 (0.14-0.21)	0.18 (0.15-0.22)	0.19 (0.16-0.23)	0.20 (0.17-0.24)	0.21 (0.18-0.24)	0.20 (0.17-0.23)	0.20 (0.17-0.23)
Dental Implant	0.13 (0.06-0.26)	0.13 (0.06-0.26)	0.17 (0.09-0.31)	0.18 (0.10-0.33)	0.17 (0.10-0.31)	0.18 (0.11-0.3)	0.18 (0.11-0.30)	0.22 (0.14-0.33)	0.23 (0.15-0.35)	0.24 (0.16-0.36)
Oral Surgery	0.34 (0.26-0.43)	0.37 (0.29-0.46)	0.37 (0.29-0.46)	0.37 (0.29-0.46)	0.37 (0.30-0.47)	0.38 (0.3-0.47)	0.37 (0.3-0.46)	0.37 (0.30-0.46)	0.36 (0.29-0.44)	0.35 (0.28-0.43)
Advanced General Dentistry	0.56 (0.44-0.71)	0.58 (0.45-0.74)	0.59 (0.46-0.74)	0.59 (0.47-0.74)	0.60 (0.48-0.75)	0.59 (0.48-0.72)	0.62 (0.50-0.76)	0.61 (0.49-0.75)	0.57 (0.45-0.72)	0.55 (0.44-0.70)
Orthodontics & Dentofacial Orthopedics	0.42 (0.37-0.47)	0.44 (0.40-0.49)	0.44 (0.39-0.49)	0.44 (0.40-0.49)	0.46 (0.42-0.51)	0.48 (0.44-0.52)	0.50 (0.46-0.54)	0.51 (0.47-0.56)	0.50 (0.46-0.54)	0.50 (0.46-0.54)
Prosthodontics	0.44 (0.39-0.51)	0.45 (0.39-0.51)	0.45 (0.4-0.52)	0.48 (0.42-0.54)	0.49 (0.44-0.55)	0.50 (0.45-0.56)	0.53 (0.48-0.58)	0.54 (0.49-0.59)	0.53 (0.48-0.58)	0.54 (0.49-0.59)
Dental Biomaterials	0.86 (0.28-2.64)	0.87 (0.28-2.68)	0.88 (0.29-2.7)	0.82 (0.26-2.56)	0.83 (0.27-2.59)	0.85 (0.27-2.62)	0.84 (0.27-2.62)	0.84 (0.27-2.62)	0.69 (0.21-2.32)	0.70 (0.21-2.33)
Family Dentistry	0.21 (0.08-0.53)	0.27 (0.12-0.63)	0.31 (0.15-0.67)	0.43 (0.27-0.7)	0.48 (0.33-0.7)	0.40 (0.29-0.56)	0.46 (0.36-0.58)	0.46 (0.38-0.55)	0.48 (0.41-0.57)	0.49 (0.41-0.58)
Preventive Dentistry	0.65 (0.28-1.51)	0.78 (0.34-1.79)	0.79 (0.35-1.79)	0.79 (0.34-1.79)	0.80 (0.35-1.82)	0.80 (0.35-1.82)	0.72 (0.31-1.70)	0.72 (0.30-1.69)	0.76 (0.32-1.79)	0.77 (0.32-1.82)
Endodontics	0.53 (0.44-0.63)	0.54 (0.45-0.63)	0.55 (0.47-0.65)	0.56 (0.48-0.65)	0.62 (0.54-0.71)	0.63 (0.56-0.71)	0.64 (0.57-0.72)	0.65 (0.58-0.72)	0.63 (0.57-0.7)	0.64 (0.58-0.71)
Periodontics	0.60 (0.50-0.72)	0.62 (0.52-0.75)	0.65 (0.55-0.78)	0.65 (0.55-0.77)	0.68 (0.59-0.80)	0.73 (0.63-0.84)	0.75 (0.66-0.87)	0.73 (0.65-0.84)	0.73 (0.65-0.83)	0.72 (0.64-0.81)
Orofacial Pain	0.62 (0.19-1.98)	0.79 (0.27-2.34)	0.80 (0.27-2.36)	0.66 (0.25-1.72)	0.85 (0.34-2.14)	0.94 (0.41-2.15)	1.02 (0.46-2.29)	1.14 (0.54-2.43)	1.01 (0.46-2.18)	1.09 (0.51-2.32)
Restorative Dentistry	0.74 (0.63-0.86)	0.77 (0.66-0.89)	0.80 (0.69-0.93)	0.81 (0.71-0.93)	0.86 (0.75-0.97)	0.89 (0.79-1.01)	0.91 (0.81-1.01)	0.94 (0.85-1.05)	0.94 (0.85-1.04)	0.93 (0.84-1.03)
Oral & Maxillofacial Radiology	0.96 (0.40-2.3)	0.96 (0.42-2.16)	0.99 (0.46-2.10)	1.18 (0.65-2.15)	1.38 (0.79-2.40)	1.23 (0.73-2.09)	1.28 (0.77-2.13)	1.32 (0.81-2.16)	1.23 (0.76-2.00)	1.26 (0.78-2.06)
Oral Medicine & Pathology	0.83 (0.55-1.26)	0.88 (0.59-1.33)	1.03 (0.7-1.51)	1.09 (0.77-1.55)	1.21 (0.89-1.66)	1.30 (0.98-1.73)	1.33 (1.02-1.74)	1.31 (1.01-1.69)	1.33 (1.04-1.70)	1.33 (1.05-1.70)
Dental Public Health	1.14 (0.74-1.76)	1.16 (0.76-1.79)	1.15 (0.75-1.76)	1.27 (0.86-1.88)	1.55 (1.08-23)	1.52 (1.09-2.11)	1.56 (1.14-2.14)	1.56 (1.16-2.1)	1.48 (1.13-1.95)	1.56 (1.19-2.04)
Paediatric Dentistry	1.19 (1.01-1.4)	1.18 (1.01-1.39)	1.21 (1.04-1.41)	1.28 (1.11-1.48)	1.37 (1.20-1.57)	1.42 (1.25-1.61)	1.42 (1.26-1.6)	1.48 (1.32-1.65)	1.45 (1.31-1.62)	1.47 (1.32-1.63)
Oral Biology	2.06 (0.88-4.84)	2.07 (0.88-4.87)	2.02 (0.89-4.59)	2.07 (0.96-4.49)	2.20 (1.05-4.59)	2.20 (1.05-4.59)	2.22 (1.06-4.64)	2.23 (1.07-4.66)	2.56 (1.17-5.56)	2.52 (1.16-5.49)

Restorative Dentistry was the only specialty that showed approximately proportional female relative representation starting in 2021 (Figure 3 and Supplementary Table S3). In addition, since 2021, the 95% CIs for the OR estimates have included 1, indicating insufficient evidence of a difference in registration between female and male dentists after adjustment for covariates. In earlier years, female dentists were relatively underrepresented and had significantly lower adjusted odds of registration in Restorative Dentistry than male dentists, as the OR estimates were consistently below 1 and the 95% CIs excluded 1.

In the remaining 15 specialties, female dentists were relatively underrepresented throughout the study period (Figure 3 and Supplementary Table S3). In addition, the adjusted ORs for these 15 specialties over the 10-years period were below 1, and the 95% CIs excluded 1, except for 3 specialties (Dental Biomaterials, Preventive Dentistry, and Orofacial Pain), in which the 95% CIs included 1, indicating insufficient evidence of a difference in registration odds between female and male dentists after adjustment for covariates. In 2025, Oral and Maxillofacial Surgery, Dental Implantology, and Oral Surgery showed the greatest disproportionality. These specialties also had the lowest adjusted ORs for female registration: Oral and Maxillofacial Surgery (OR = 0.20, 95% CI: 0.17-0.23), Dental Implantology (OR = 0.24, 95% CI: 0.16-0.36), and Oral Surgery (OR = 0.35, 95% CI: 0.28-0.43). Most of the 15 specialties demonstrated varying degrees of increase in female relative representation over the 10-year period. The most pronounced increases were observed in Family Dentistry and Orofacial Pain. Female relative representation did not change substantially over the study period in Preventive Dentistry and Oral and Maxillofacial Surgery. In contrast, the magnitude of female underrepresentation increased in 2 specialties: Advanced General Dentistry and Dental Biomaterials.

## Discussion

This study aimed to assess gender representation in the dental workforce in Saudi Arabia from 2016 to 2025, including temporal patterns of segregation and disparities across specialties and professional ranks. Although the overall representation of female dentists increased steadily during the study period, showing a strong linear upward trend, female dentists in Saudi Arabia did not reach numerical parity. Female dentists were also relatively underrepresented in higher professional ranks throughout the study period, despite noticeable improvement over time. This pattern was consistent with findings from proportional-odds ordinal logistic regression models, which showed that female dentists had consistently lower odds than male dentists of being represented in higher professional ranks across all years, even after adjustment for age group, nationality, and country of qualification. However, these disparities appeared to narrow gradually over time. Across dental specialties, female dentists were relatively underrepresented in most specialties during the study period, with the greatest underrepresentation observed in the surgical specialties. In contrast, female dentists were relatively overrepresented mainly in nonclinical specialties, as well as in paediatric dentistry. These findings on relative representation were largely consistent with the findings from the adjusted weighted multinomial logistic regression models.

The lower number of female dentists compared with male dentists during the study period may partly reflect historical differences in access to dental education. In Saudi Arabia, female sections in some dental schools were introduced later than male sections, and female student admission capacity may have been lower in earlier years.^21^ Other barriers to workforce entry for female dentists in Saudi Arabia may help explain why they remained a minority throughout the 10-year study period (2016–2025). One such barrier is the conflict between family responsibilities and work obligations.^5^^,^^6^ This may reflect female dentists’ need or tendency to prioritise childcare responsibilities and household duties over their dental careers; however, it may also reflect societal pressures stemming from traditional Saudi gender norms.^5^ Such pressures may also affect unmarried female dentists, as entering the workforce may reduce a woman’s perceived likelihood of marriage because of cultural notions that a busy, successful woman may be less able to fulfill her home and family responsibilities.^5^ Another barrier was the historical ban on women driving in Saudi Arabia, which was lifted in 2018. Before that, female dentists had to rely on male relatives or spend a large portion of their income on private drivers.^5^^,^^6^ Coupled with the lack of reliable public transportation, this severely limited their mobility and employment options.^22^

Even though the underrepresentation of female dentists identified in Saudi Arabia in the present study was also observed in many other countries, a small number of countries had female-majority dental workforces during the study period.^23^^,^^24^ Notable examples include Finland, Russia, Latvia, and Lithuania, where nearly 4 in 5 dentists were female.^23^ The high representation of female dentists in some of these countries may be partly explained by cultural values and gender norms, which may have contributed to the perception of care-oriented professions such as dentistry as inherently female.^25^ Another possible explanation is the presence of a well-established and expansive public healthcare system.^25^^,^^26^ For example, the Public Dental Service in Finland is often highly attractive to female dentists because it is perceived to better support work–life balance through job stability, predictable daytime working hours, and generous maternity leave benefits.^25^ This may also help explain the high female dentists representation in some former communist countries, where the feminisation of the public health sector has been linked to state policies that combined low wages with long maternity leave provisions and extensive public childcare support.^26^

A notable finding from this study is the steady increase in the number of female dentists in the Saudi dental workforce over the study period, which forms part of a broader global phenomenon.^27^ Important contextual factors that may be associated with this increase within the Saudi dental workforce include policy and legislative reforms introduced in Saudi Arabia over the past 2 decades to expand women’s participation in the national workforce. For example, in 2004, the Saudi government passed Resolution 120, which aimed to expand and promote female employment across the public and private sectors.^28^ In addition, under Saudi Vision 2030, launched in 2016, several programs and reforms were implemented to further increase female participation in the national workforce.^29^^,^^30^ These policies and legislation were also accompanied by a substantial expansion in female enrollment in dental education.^31^ For example, the establishment and expansion of women-only dental education institutions, such as Princess Nourah bint Abdulrahman University, in 2012 may have helped increase opportunities for females to study dentistry and may therefore have contributed to narrowing the gender gap over time.^32^ The increase in female representation in the dental workforce may also be interpreted in light of previous studies reporting Saudi women’s preference for dentistry as a career.^22^^,^^33^ Saudi women have been reported to perceive dentistry as a profession that offers the opportunity to help others, relieve pain, and improve patients’ appearance.^22^^.^^33^

Throughout the study period, female dentists tended to be concentrated in the lower professional ranks and consistently had lower odds of being represented in higher professional ranks than their male counterparts, even after adjustment for several potential confounders. In the SCFHS system, progression to higher professional ranks depends on completing postgraduate qualifications.^18^ which previous evidence suggests female dentists may be less likely to pursue.^8^ This form of vertical segregation in the dental workforce has been observed worldwide.^8^ Evidence from medical education provides insights into factors that may contribute to such patterns. A systematic review of 34 studies identified work–life balance and family considerations, gender discrimination, negative perceptions of male-dominated specialties, and limited access to female role models as recurring influences on women’s decisions regarding medical specialty training.^34^ Previous studies from the dental field have likewise suggested that the lack of flexible working arrangements and family-friendly policies in many residency programs may contribute to this pattern by placing female dentists in a position of conflict between pursuing postgraduate qualifications and childbearing during key career-development years.^35^ Consistent with this, previous evidence showed that 60.7% of female specialists completed their postgraduate education before having children, compared with only 34.3% of male specialists.^27^ Another factor suggested in the literature is the limited availability of female mentors and role models, who may help women overcome barriers along the challenging pathway of postgraduate training.^36^

Even though the previously mentioned factors that may prevent female dentists from completing postgraduate training have also been reported among women in dentistry in Saudi Arabia, additional contextual factors may further exacerbate this form of vertical segregation.^5^^,^^22^^,^^37^ For example, women in Saudi Arabia were previously not permitted to travel abroad without a male guardian, which may have restricted their access to postgraduate training opportunities outside the country.^37^ In addition, even within Saudi Arabia, many training positions may be located outside major cities, and relocating away from family may impose substantial cultural and social challenges, particularly for single women.^22^ However, vertical gender segregation in the Saudi dental workforce appeared to narrow over the 10-year study period, a trend that may be interpreted in relation to broader policy reforms and shifting social norms.^22^

Another notable finding in this study was the uneven distribution of female dentists across specialties. Female dentists in Saudi Arabia were relatively overrepresented in a limited number of specialties, particularly nonclinical specialties and paediatric dentistry. In contrast, they were relatively underrepresented in most other specialties, with the greatest underrepresentation observed in surgical specialties. This pattern of horizontal segregation persisted even after adjustment for several potential confounders. A similar pattern of horizontal segregation has also been observed in many other countries.^16^^,^^23^^,^^38^^,^^39^ The specialty choices of female dentists may reflect relatively stronger care-oriented and altruistic values.^40^^,^^41^ Previous research suggests that female dentists place greater value on interpersonal relationships, compassion, and the humanistic aspects of clinical practice.^40^^,^^42^ These factors may help explain their greater preference for specialties such as paediatric dentistry and dental public health.^43^ In contrast, additional environmental factors may further explain the pronounced relative underrepresentation in surgical specialties. Many female dentists have described the surgical work environment as a male-dominated culture marked by gender discrimination and hostility.^37^ For example, female dentists have reported facing negative stereotypes from their male colleagues, who assumed that women lacked the physical and emotional resilience required for surgical practice.^44^ More disturbingly, surveys of female surgeons have revealed that 38% experienced sexual harassment during residency, and 48% reported gender bias in their practice environment.^44^ These may reflect broader workplace experiences within surgical training. A systematic review reported that bullying, discrimination, harassment, sexual harassment, and fear of retaliation were widely documented among surgical residents, with women disproportionately affected by several of these experiences.^45^

Even though this study did not attempt to forecast future gender patterns in the Saudi dental workforce, its findings may provide some insight into how these patterns could evolve over time. One notable trend that may continue is the growing number of female dentists in the workforce, accompanied by a narrowing of horizontal and vertical gender segregation, although the pace of this trend appears to have slowed after 2022. This may reflect a broader global phenomenon often described as the feminisation of dentistry.^27^ Several implications of this phenomenon have been proposed and may also be relevant to the Saudi dental workforce context. For instance, female dentists have been reported to work, on average, fewer hours over the course of their careers than their male counterparts.^16^^,^^27^^,^^46^ Accordingly, if female dentists become the majority, workforce planning may need to place greater emphasis on capacity planning rather than simple dentist-to-population ratios.^23^ This is consistent with recent access-to-care research suggesting that conventional workforce indicators may not fully capture effective access to dental care.^47^ Another proposed implication of the feminisation of dentistry is a possible shift away from traditional solo private practice ownership toward employee and associate roles.^23^^,^^27^ a trend for which evidence is emerging in several European countries where female dental students outnumber males by approximately 2:1.^48^ If sustained, such a shift could influence patterns of dental practice ownership and potentially reduce levels of dental entrepreneurship.^27^ Finally, the feminisation of dentistry may contribute to existing urban–rural disparities in dental care, as female dentists have been reported to be less likely than their male counterparts to practice in rural or remote communities.^16^^,^^36^ Therefore, targeted policies and regulatory measures may be needed to support and incentivise practice in underserved areas.^27^

Several limitations of this study should be considered when interpreting its findings. For instance, several potentially important covariates were not adjusted for in the regression models because relevant data were unavailable. These may include years since graduation or professional experience, employment sector, academic versus private practice roles, practice location, workload intensity, part-time versus full-time status, leadership appointments, marital status, and other family-related factors. These variables may influence both professional-rank distribution and specialty representation; therefore, the possibility of residual confounding should be acknowledged. In addition, the study relied on repeated annual aggregated registry data rather than individual-level longitudinal records. Consequently, the career progression of individual dentists could not be tracked over time. Another limitation was that registration of training residents with the SCFHS was not mandatory before 2017; therefore, data for this group were unavailable before that year. Furthermore, several specialties included a relatively small number of registered dentists, resulting in reduced statistical precision, as reflected by wide 95% CIs. For some specialties, such as Oral Biology, Dental Biomaterials, Preventive Dentistry, and Orofacial Pain, the 95% CIs for the estimated odds ratios included 1, indicating insufficient evidence of a difference in registration odds between female and male dentists. Therefore, findings related to these specialties should be interpreted with caution. Finally, as with all administrative registry data, the possibility of inaccuracies or delays in data entry and updating cannot be excluded.

Future research should address the main limitations of this study by using individual-level data and incorporating a broader range of relevant covariates. Further research is also needed to forecast future trends in the gender structure of the Saudi dental workforce. In addition, future studies should examine the factors and causes underlying gender disparities and patterns of horizontal and vertical segregation within the Saudi dental workforce.

## Conclusion

The Saudi dental workforce has exhibited persistent gender disparities over the past decade, including patterns of both horizontal and vertical gender segregation. Female dentists remained a minority throughout the study period and had lower odds than male dentists of being represented in higher professional ranks and of being registered in most specialties, particularly surgical specialties. However, these disparities and segregation patterns appeared to become less pronounced over time. The evolving gender composition of the dental workforce in Saudi Arabia may have important implications for workforce planning, service organisation, and access to dental care. Future research is needed to identify the factors underlying these patterns and to forecast future gender trends in order to inform policies aimed at improving gender equity within the Saudi dental workforce and ensuring that workforce planning remains responsive to the needs of Saudi Arabia’s growing population.

## Author contributions

*Concept and design*: All authors.

*Acquisition, analysis, or interpretation of data*: All authors.

*Drafting of the manuscript:* Fahad BaHammam and Fathima Farook.

*Critical revision of the manuscript for important intellectual content:* All authors.

*Statistical analysis:* Fathima Farook.

## Data availability

The dataset used and analysed during the current study is available from the corresponding author on reasonable request.

## Declaration of competing interest

None disclosed.

## Appendix Supplementary materials

Image, application 1
