# Supplementary material for: National Trends in Gender Representation, Segregation, and Disparities in the Saudi Dental Workforce, 2016–2025
Source: Int Dent J. 2026 Aug 17;76(5):111091. doi: 10.1016/j.identj.2026.111091 (PMC13507748; doi:10.1016/j.identj.2026.111091)
Supplement: Supplementary file 1 [file mmc1.docx]

| Supplementary Table S1. Mapping of original Saudi Commission for Health Specialties (SCFHS) specialty labels to harmonized specialty categories | |
| --- | --- |
| Harmonized categories | Original SCFHS specialty labels |
| Dental Implant | Dental Implant  Implant Dentistry  Reconstruction and Dental Implant  dental implants |
| Oral & Maxillofacial Surgery | Oral and Maxillofacial Surgery  Maxilofacial Surgery and implant  Plastic Surgery  Cosmatic Facial Surgery  Maxillofacial Oncology and Microvascular Reconstruction  Faical Aesthetic and Cosmetic surgery  Head and Neck Cancer-Microvascular Reconstruction  Orthognathic and Facial Plastic Surgery  Orthognathic surgery  Esthetic Facial Surgery and TMJ  Pediatric Oral and Maxillofacial Surgery |
| Oral Surgery | Oral Surgery  Dental Surgery |
| Family Dentistry | Family Dentistry |
| Orthodontics & Dentofacial Orthopedics | Orthodontics and Dentofacial Orthopedics  Orthodontics  Craniofacial Orthodontic  Pediatric Orthodontics |
| Prosthodontics | Prosthodontics  Maxillofacial Prosthdontics  Prosthodontics and Dental Material  Maxillofacial Prosthetics and Oncologic Dentistry  Maxillofacial Prosthodontics  Oral Oncology and Maxillofacial Prosthodontics |
| Advanced General Dentistry | Advanced General Dentistry |
| Endodontics | Endodontics  Endodontic  Advanced Endodontics |
| Preventive Dentistry | Preventive Dentistry |
| Orofacial Pain | Orofacial Pain  Orofacial Pain and T.M.D  Temporomandibular Joint Disorders  Temporomadibular Joint |
| Periodontics | Periodontics  Oral Biology/Periodontics |
| Dental Biomaterials | Dental Biomaterials |
| Restorative Dentistry | Restorative Dentistry  Conservative Dentistry |
| Oral & Maxillofacial Radiology | Oral & Maxillofacial Radiology |
| Oral Medicine & Pathology | Oral Medicine  Oral Diagnosis  Oral and Maxillofacial Pathology |
| General Dentistry | General Dentistry  Dental  Clinical Dentistry |
| Dental Public Health | Dental Public Health  Public Health  Epidemiology  Public Health- Infection Control  COMMUNITY MEDICINE AND DENTISTRY |
| Pediatric Dentistry | Pediatric Dentistry  Pediatrics |
| Oral Biology | Oral Biology |

| Supplementary Table S2: Female relative representation, based on LDI values, across professional ranks in Saudi Arabia, 2016–2025; LDI = 1 indicates proportional representation, LDI > 1 female overrepresentation, and LDI < 1 underrepresentation | | | | | | | | | | |
| --- | --- | --- | --- | --- | --- | --- | --- | --- | --- | --- |
| Rank | 2016 | 2017 | 2018 | 2019 | 2020 | 2021 | 2022 | 2023 | 2024 | 2025 |
| Consultant | 0.6888527 | 0.6801468 | 0.7085638 | 0.7282819 | 0.7428622 | 0.7436116 | 0.7484564 | 0.7711746 | 0.7964533 | 0.8003293 |
| General Dentist | 1.1072826 | 1.1041241 | 1.1023324 | 1.1036177 | 1.0990573 | 1.0979019 | 1.0960933 | 1.0966692 | 1.0985353 | 1.0983209 |
| Registrar | 0.5663623 | 0.5719264 | 0.5725659 | 0.5742828 | 0.5816532 | 0.5873937 | 0.6049932 | 0.6162390 | 0.6129941 | 0.6125818 |
| Resident | 0.8803167 | 0.8869815 | 0.8912118 | 0.8669198 | 0.8403820 | 0.8308727 | 0.8420882 | 0.8282137 | 0.8274041 | 0.8305848 |
| Senior Registrar | 0.7005095 | 0.7549718 | 0.7734010 | 0.7831699 | 0.8165210 | 0.8443812 | 0.8940870 | 0.9127314 | 0.9545018 | 1.0056052 |
| Training Resident | *NA* | 1.5885612 | 1.0829717 | 1.1755736 | 1.3043748 | 1.2649796 | 1.2357134 | 1.2035944 | 1.2283664 | 1.1769818 |

| Supplementary Table S3: Female relative representation, based on LDI values, across dental specialties in Saudi Arabia, 2016–2025; LDI = 1 indicates proportional representation, LDI > 1 female overrepresentation, and LDI < 1 underrepresentation | | | | | | | | | | |
| --- | --- | --- | --- | --- | --- | --- | --- | --- | --- | --- |
| Specialty | 2016 | 2017 | 2018 | 2019 | 2020 | 2021 | 2022 | 2023 | 2024 | 2025 |
| Dental Implant | 0.2237772 | 0.2186384 | 0.2699099 | 0.2937022 | 0.2741966 | 0.2747737 | 0.2766095 | 0.3151209 | 0.3351715 | 0.3455956 |
| Oral & Maxillofacial Surgery | 0.2522072 | 0.2437504 | 0.2512459 | 0.2760333 | 0.2929451 | 0.3059156 | 0.3249152 | 0.3391153 | 0.3328989 | 0.3416385 |
| Oral Surgery | 0.4675623 | 0.4919227 | 0.4883238 | 0.4852664 | 0.4854628 | 0.4808539 | 0.4728093 | 0.4711444 | 0.4665391 | 0.4598435 |
| Family Dentistry | 0.3869481 | 0.4077780 | 0.4300035 | 0.6045132 | 0.6796986 | 0.6370704 | 0.6835492 | 0.6767476 | 0.7009714 | 0.7108502 |
| Orthodontics & Dentofacial Orthopedics | 0.5963473 | 0.6103689 | 0.5997205 | 0.6075074 | 0.6287251 | 0.6449034 | 0.6646129 | 0.6855952 | 0.6887553 | 0.6926137 |
| Prosthodontics | 0.6103084 | 0.6039485 | 0.5997997 | 0.6276700 | 0.6410441 | 0.6449409 | 0.6724630 | 0.6883063 | 0.6862960 | 0.6949639 |
| Advanced General Dentistry | 0.7738962 | 0.7685047 | 0.7544502 | 0.7478081 | 0.7432767 | 0.7334178 | 0.7482445 | 0.7384044 | 0.7033974 | 0.6881716 |
| Endodontics | 0.7104221 | 0.7028916 | 0.7091382 | 0.7202201 | 0.7714870 | 0.7808650 | 0.7928115 | 0.8067443 | 0.8048545 | 0.8126794 |
| Preventive Dentistry | 0.7429404 | 0.8220804 | 0.8097296 | 0.8005483 | 0.7896861 | 0.7789834 | 0.7133613 | 0.7100723 | 0.7383489 | 0.7384154 |
| Orofacial Pain | 0.6633396 | 0.7611856 | 0.7497496 | 0.6671235 | 0.7677504 | 0.8114410 | 0.8560336 | 0.9129502 | 0.8648253 | 0.9098333 |
| Periodontics | 0.7609259 | 0.7625522 | 0.7717250 | 0.7722774 | 0.7919574 | 0.8210419 | 0.8422725 | 0.8377731 | 0.8464382 | 0.8410622 |
| Dental Biomaterials | 0.8929572 | 0.8782911 | 0.8650957 | 0.8552866 | 0.8436818 | 0.8322472 | 0.8231092 | 0.8193142 | 0.7075843 | 0.7076481 |
| Restorative Dentistry | 0.9126228 | 0.9065806 | 0.9067369 | 0.9067699 | 0.9283584 | 0.9452483 | 0.9467870 | 0.9732888 | 0.9748840 | 0.9730162 |
| Oral & Maxillofacial Radiology | 0.9950094 | 0.9514820 | 0.9639638 | 1.0613329 | 1.1182919 | 1.0432813 | 1.0525003 | 1.0651085 | 1.0455350 | 1.0614722 |
| Oral Medicine & Pathology | 0.9042366 | 0.9181311 | 0.9788398 | 1.0185092 | 1.0700354 | 1.1093813 | 1.1233020 | 1.1216245 | 1.1369052 | 1.1364461 |
| Dental Public Health | 1.0779269 | 1.0621194 | 1.0361708 | 1.0794879 | 1.1687067 | 1.1490751 | 1.1564429 | 1.1543634 | 1.1506511 | 1.1794135 |
| Pediatric Dentistry | 1.1457684 | 1.1189076 | 1.1114323 | 1.1468371 | 1.1771543 | 1.1922794 | 1.1919686 | 1.2193215 | 1.2195762 | 1.2261230 |
| Oral Biology | 1.3719069 | 1.3493745 | 1.3120618 | 1.3177749 | 1.3161436 | 1.2983056 | 1.2840504 | 1.2781302 | 1.3646269 | 1.3647500 |
